# Whole-exome sequencing in an isolated population from the Dalmatian island of Vis

**DOI:** 10.1038/ejhg.2016.23

**Published:** 2016-04-06

**Authors:** Ana Jeroncic, Yasin Memari, Graham RS Ritchie, Audrey E Hendricks, Anja Kolb-Kokocinski, Angela Matchan, Veronique Vitart, Caroline Hayward, Ivana Kolcic, Dominik Glodzik, Alan F Wright, Igor Rudan, Harry Campbell, Richard Durbin, Ozren Polašek, Eleftheria Zeggini, Vesna Boraska Perica

**Affiliations:** 1 Department of Research in Biomedicine and Health, University of Split School of Medicine, Split, Croatia; 2 Wellcome Trust Sanger Institute, Hinxton, Cambridge, UK; 3 Department of Mathematical and Statistical Sciences, University of Colorado, Denver, CO, USA; 4 MRC Human Genetics Unit, Institute for Genetics and Molecular Medicine, University of Edinburgh, Edinburgh, UK; 5 Department of Public Health, University of Split School of Medicine, Split, Croatia; 6 Centre for Global Health Research, University of Edinburgh, Edinburgh, UK; 7 Department of Medical Biology, University of Split School of Medicine, Split, Croatia

## Abstract

We have whole-exome sequenced 176 individuals from the isolated population of the island of Vis in Croatia in order to describe exonic variation architecture. We found 290 577 single nucleotide variants (SNVs), 65% of which are singletons, low frequency or rare variants. A total of 25 430 (9%) SNVs are novel, previously not catalogued in NHLBI GO Exome Sequencing Project, UK10K-Generation Scotland, 1000Genomes Project, ExAC or NCBI Reference Assembly dbSNP. The majority of these variants (76%) are singletons. Comparable to data obtained from UK10K-Generation Scotland that were sequenced and analysed using the same protocols, we detected an enrichment of potentially damaging variants (non-synonymous and loss-of-function) in the low frequency and common variant categories. On average 115 (range 93–140) genotypes with loss-of-function variants, 23 (15–34) of which were homozygous, were identified per person. The landscape of loss-of-function variants across an exome revealed that variants mainly accumulated in genes on the xenobiotic-related pathways, of which majority coded for enzymes. The frequency of loss-of-function variants was additionally increased in Vis runs of homozygosity regions where variants mainly affected signalling pathways. This work confirms the isolate status of Vis population by means of whole-exome sequence and reveals the pattern of loss-of-function mutations, which resembles the trails of adaptive evolution that were found in other species. By cataloguing the exomic variants and describing the allelic structure of the Vis population, this study will serve as a valuable resource for future genetic studies of human diseases, population genetics and evolution in this population.

## Introduction

Recent advances in genotyping and sequencing technologies have opened a route to a new dimension of population studies, enabling the development of clear insight into the past of any population and corroboration of existing evidence from the domains of palaeontology, archaeology and historical evidence with unprecedented validation and precision. This type of analysis is interesting not only on the global scale,^1^ but also on a local scale, particularly in the case of special and isolated populations. Such populations may retain their genetic isolation and uniqueness due to a number of possible factors, including geographical, ethnic or linguistic barriers, and have been estimated to encompass over 11.5 million individuals in Europe alone.^2^

The Croatian Adriatic islands are geographically isolated, habitat-unique localities characterized by distinctive population histories, which include varying founding times and consequent population age, severe plague bottlenecks and massive waves of emigration due to deteriorating economical conditions.^3^ The genetic structure was also affected by historic events, such as near annihilation of the island populations in the conflicts with the ancient Roman Empire, a shift caused by the massive Slavs arrival in ~700 ad and clashes with the Ottoman Empire.^3, 4, 5, 6^ Genetic studies revealed a reduction of haplotype diversity in Vis compared to an outbread population from Scotland and presented a founder effect in Vis mtDNA sequences.^7^ Besides drift and increased homogeneity, isolated populations also tend to harbour unique rare variants,^8^ making them very useful tools in genetic association studies.^9^ These properties have contributed to the development of the 10 001 Dalmatians resource (http://www.mefst.hr/default.aspx?id=826), the largest research-oriented biobank in Croatia, now commonly used in genetic studies worldwide.^10^

The aim of this study is to perform an exhaustive exploratory analysis of the exomic structure of the modern-day population of the island of Vis. By cataloguing exomic variants, investigating their frequencies and functional effects, and examining autozygosity, we describe the allelic architecture of this isolated population, providing the basis for a valuable resource for future genetic studies focusing on disease susceptibility, population genetics and evolution.

## Materials and Methods

### Participant recruitment and sample collection

We conducted exome-wide sequencing of 193 individuals from the isolated population of the island of Vis, Croatia. The sample for this study was based on the initial cohort of 1026 participants, which were initially recruited in the CROATIA-Vis study (the 10 001 Dalmatians project) between 2003 and 2004.^11^ The participants were recruited on the basis of vital registries, postal invitations and other means of invitations. In total, 193 participants (38% men) were selected for the purpose of this study on the basis of three criteria. The first criterion was that a participant originated from the island of Vis, which was verified by the Parish records that were reconstructed for the period of 1850 onwards, and later corroborated by the genealogical records provided by the subjects. Second, we used the participants whose DNA was successfully extracted and who were not identified as genetic outliers in the initial CROATIA-Vis study by principal components analysis of genome-wide, Illumina HumanHap300 (San Diego, CA, USA) genotypes. Last, we used ANCHAP, a method for detecting identity by descent in isolated populations, to select a sample of participants which maximised the whole sample haplotypes representation.^7^ Mean genomic kinship of the sample was 0.002, as estimated by the KING algorithm.

All subjects were asked to provide written consent, after being informed on the study goals and main approaches, in accordance with the Declaration of Helsinki. The study was approved by the ethics committees of the University of Zagreb (No. 018057) and the University of Split School of Medicine (No. 2181-198-A3-04110-11-0008), Croatia and the Multi-Centre Research Ethics Committee for Scotland (No. 01/0/71).

### Exome capture and sequencing

Sequencing was performed at the Wellcome Trust Sanger Institute, Hinxton, Cambridge, UK. The exomes were captured from blood genomic DNA, using SureSelect Human All Exon SeqCap pulldown technology and were sequenced with 75 bp paired-end reads on Illumina HiSeq platform according to manufacturer's protocol. The capture kit used for exome enrichment was Agilent's SureSelect Human All Exon 50 Mb (Agilent, Santa Clara, CA, USA), targets 51.8 Mb of human genome by design and encompasses unified set of coding exons annotated on August, 2010 by GENCODE or CCDS databases (including 10 bp of flanking sequence for each consensus coding DNA sequence). The probe design also includes small non-coding RNA regions annotated by miRBase v.13 (Manchester, UK) and Rfam (Hinxton, UK) databases, and overall, reports a 100% breadth of coverage.

Compared to the CCDS database, the total of 653 828 probes tile to 97% of targeted bases. If regions captured upstream and downstream of targets are considered, the kit targets 90.5 Mb of genome, providing additionally a broad coverage of non-coding DNA in exon-flanking regions (promoters and untranslated regions (UTRs)).

### Variant calling and annotation

Details of the workflow used for single nucleotide variant (SNV) calling on the exome data are given in Supplementary material. Called SNVs were annotated with dbSNP137 rsIDs and 1000Genomes super population allele frequencies that were extracted from the final 1000Genomes Phase 1 integrated (v3) callset. Functional annotations were called with the Ensembl Variant Effect Predictor v2.8 against Ensembl 70, which provided coding consequence predictions and SIFT, PolyPhen and Condel annotations as well as GERP conservation scores (http://www.ensembl.org/info/docs/tools/vep/index.html).

The same calling and annotation protocol was also applied to the Generation Scotland UK10K_OBESITY_GS whole-exome data, that was used for comparison with Vis dataset (UK10K-GS—release 2012-11-27 variant dataset; after the initial quality control dataset included *n*=377 samples from Scotland ascertained on the basis of body mass index (BMI)>40 or pedigrees discordant for BMI). Variants in the UK10K-GS dataset were generated by whole-exome sequencing (WES) as part of the UK10K project that used the same targeted regions, sequencing protocols and downstream bioinformatics pipeline as our study.

The generated WES data have been submitted to the European Genome-phenome Archive (https://www.ebi.ac.uk/ega/home) with the accession numbers EGAS00001000336 and EGAD00001001387.

### Performance of whole-exome sequencing

Sequencing depth and coverage of targeted regions were calculated using Samtools' 0.1.19 bamcheck (v2012-09-04, http://samtools.sourceforge.net).

We collected high-coverage exon-capture data for 193 samples with a median of 99.9 (range, 71.6–170.1) million reads per subject, and median raw depth for the SureSelect target regions of 110 ×. Median insert size across all samples was 181 (173–203) bp per sample. On average, 99.6% (99.2–99.7%) of per subject reads were mapped to the human reference genome, with reads in the target regions mapped to 100%. After removal of PCR duplicates, 90.7% (84.8–93.8%) of per subject reads were retained. Among those uniquely mapped, 85.1% (79.2–93.9%) of per subject reads were within the target regions.

The median value of aligned-read depth on the target regions per subject was 110 × (78–193) with 51.5 Mb (99.5% of target regions) covered by at least 30 ×, and 39.9 Mb (77.2%) covered by at least 50 ×. For sequences falling outside the target but within the calling regions, 29.2 Mb (75.3% of out-target calling region) were covered by at least 30 × read and 15.6 Mb (40.2%) were covered by at least 50 × reads. The analysis of aggregate on target *T*_i_/*T*_v_ ratio implied that the calling pipeline produced a good quality variant callset (Supplementary material, *T*_i_/*T*_v_ ratio).

Standard sample and SNV quality controls were performed on the Vis and the UK10K-GS multi-sample called exome-sequence datasets, and are described in detail in Supplementary material. Close relatives were removed from both samples based on pairwise identity by descent sharing. Only bi-allelic SNVs were included in the study.

### General description of Vis exome sequence

We examined the distribution of all SNVs according to internal, sample specific allele frequency categories. We also examined the proportion of functional effects in each allele frequency category. For description purposes we used the most deleterious effect of the variant according to severity estimated by Ensembl (http://www.ensembl.org/info/docs/variation/predicted_data.html). For the data representation in the main text we have summarised Ensembl consequence annotations into fewer categories (Supplementary Table 1).

We compared Vis exome data with the UK10K-GS WES dataset, NHLBI GO Exome Sequencing Project (NHLBI) (http://evs.gs.washington.edu/EVS/), NCBI Reference Assembly dbSNP (dbSNP) (http://www.ncbi.nlm.nih.gov/projects/SNP/) as well as 1000Genomes (http://www.1000genomes.org/data#DataAvailable) and Exome Aggregation Consortium datasets (ExAC) (http://exac.broadinstitute.org/) in order to identify novel variants not reported in the reference datasets. We uniquely identified variants using chromosome, base pair position and an allele identity in each dataset before comparison. All variants were on hg19, build37.

To calculate d*N*/d*S* ratios we determined the number of non-synonymous (missense, stop_lost, stop_gained or initiator_codon variants) and synonymous (synonymous and stop_retained variants) substitutions and adjusted the values for multiple substitutions. Only substitutions within known protein-coding DNA sequence were analysed.

Autozygosity was detected through runs of homozygosity (ROH) on a set of common, independent SNVs. Owing to reduced SNV density in such set (~50 k), only long ROHs (>5 Mb) were analysed. Calls were made in PLINK v1.07 (http://pngu.mgh.harvard.edu/~purcell/plink/) using parameters optimised for detection of ROHs in WES sequences.^12^ ROH hotspots and coldspots were identified if the SNP-wise ROH frequency was above or below the 95%th percentile of the level of sharing, respectively.

### Loss of function variants

SNVs predicted by the Ensembl Variant Effect Predictor as stop-gained (nonsense) or splice site-disrupting (splice donor or acceptor) SNVs were defined as loss of function (LoF) variants. In total 1775 putative LoF variants were obtained in Vis. Potential LoF variants that were likely due to reference errors or annotation artefacts were filtered. SNVs were marked as false positives if the inferred LoF allele was also the ancestral state (73 SNVs in Vis and 115 in UK10K-GS) indicating that a gain-of-function allele was the recent mutation on a site. We also excluded LoF SNVs (24 in Vis and 17 in UK10K-GS) that were identified as major allelic variant (MAF>0.5) in our samples, and in all super populations from the 1000Genomes project: European (EUR), Asian (ASN), African (AFR), Ad Mixed American (AMR). At these sites the reference genome differed from majority of analysed humans indicating a probable reference genome error. In example, at these sites we observed extremely high frequency of homozygous LoF variants (0.26–0.89) (Supplementary Table 2). After filtering, there were 1678 remaining LoF variants in Vis, and 3149 in the UK10K dataset.

The analysis of over-representation of Gene Ontology (GO) annotations and the pathway analysis were performed on a set of genes containing LoF variants using ConsensusPathDB-human database (http://consensuspathdb.org/).^13^ False discovery rate *q*-value of 0.1 and a *P*-value of 0.05 were used to identify over-represented functional groups.

## Results

### General description of Vis exome sequence

Overall, the performance of whole-exome sequencing with high percentage of reads uniquely mapped to target regions, with aligned-read depth on target regions of 110 ×, and 51.5 Mb (99.5%) of target regions covered by at least 30 × ; indicated efficient targeted sequencing.

We found 290 577 SNVs of which 65% were singletons, low frequency or rare (Table 1, more detailed category definitions are provided in Supplementary Table 3). Rare variants (MAF⩽0.01) were the most prevalent in both LoF and nonsynonymous variants, with the percentage of rare SNVs among LoF variants significantly outnumbering the corresponding percentage among nonsynoymous SNVs. On contrary, the percentages of both low (0.01<MAF⩽0.05), and common (MAF>0.05) frequency variants among nonsynonymous SNVs was significantly higher than among LoF SNVs. (Supplementary Figure 2a, 95% confidence interval (CI)).

Relative to UK10K-GS, the Vis sample shows a depletion of rare variants and excess of low frequency and common variants (Supplementary Figure 2b). The comparison of potentially deleterious variants (non-synonymous and LoF) showed the same pattern, that is, a decrease of rare, and an excess of low and common frequency variants in Vis (Supplementary Figure 2b). This difference was not the consequence of different sample sizes. When we randomly re-sampled (*n*=100) the same number of individuals from each population and compared allele frequencies of variants shared by both populations, again a decrease in proportion of shared-rare variants (p 7.1 × 10^−27^), and increase in proportion of shared low-frequency variants (p 7.0 × 10^−20^) were observed in Vis (Supplementary Figure 2c). Moreover, frequency of shared variants declared as rare or low frequency in Vis was significantly increased compared to the same variants in UK10K-GS. Median difference in allele frequency per shared variant was 0.0008 for rare and 0.003 for low-frequency variants, which corresponds in both cases to 8% of the total frequency range of particular frequency category (one sample *U*-test for median of differences against zero, *P*⩽3.2 × 10^−20^).

**Figure 1:**
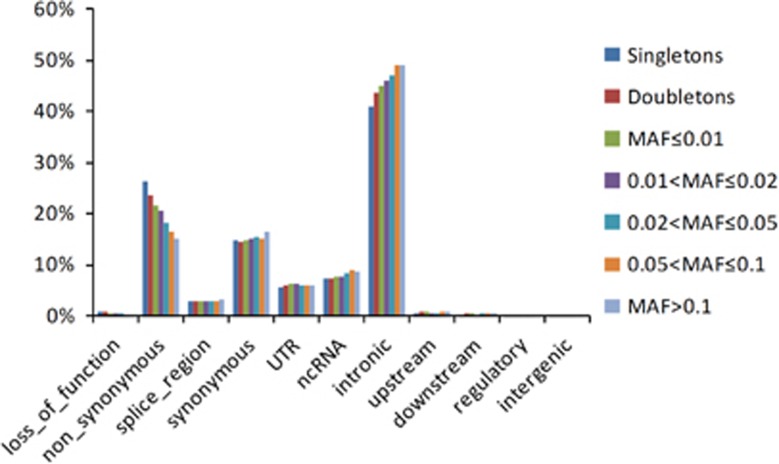
Proportion of functional effects by allele frequency categories.

The majority of detected variants in Vis are intronic (45%) (Figure 1; more detailed category definitions are provided in Supplementary Figure 3) pointing out that our baits also captured some of the intronic regions that surround exons. Intron SNVs were positioned predominantly (83.3%) within 200 bp from the nearest exon boundary with a median distance of 89 bp (interquartile range 53–132). If we exclude intronic regions then, consistently with previous reports, non-synonymous variants are the most common, followed by synonymous variants and of these the majority are singletons and rare variants (Table 1). Aggregate site frequency spectra by variant functional consequence are shown in Supplementary Figure 4.

The ratio of substitution rates at non-synonymous and synonymous sites for all variants was 1.20; 1.57 for rare and 1.16 for low-frequency variants and 0.86 for common variants. The d*N*/d*S* ratios revealed that non-synonymous substitutions were prevalent in rare and low-frequency variants, whereas the trend was reversed for common variants. Consistent with purifying selection in large populations these values were somewhat lower in UK10K-GS dataset: d*N*/d*S*=1.11 for all variants; and 1.38, 1.09 and 0.85 for rare, low frequency and common variants, respectively. In line with this finding, median inbreeding coefficient F_IT_ for major histocompatibility complex region showed reduced heterozygosity in Vis: −0.021, compared to UK10K-GS: −0.036.

The average genome-based kinship in Vis sample was low (0.002) indicating that, as expected, unrelated individuals were selected in the study. On the other hand, the average individual autozygosity in Vis that was derived from long ROHs (>5 Mb) was consistent with second-cousin relationship, F_ROH>5Mb_ 0.017, standard error of mean (SEM) 0.004.^14^ Moreover, in comparison to UK10K-GS (F_ROH>5Mb_ 0.001, SEM 0.0007), a significantly higher proportions of individuals with long ROHs were found in Vis (1 versus 11% in Vis, χ^2^, *P*=4.34 × 10^−7^). Median lengths per affected individual in Vis of total and per segment ROH length were 10 Mb (range, 7–35) and 9 Mb (7–22), respectively, whereas the corresponding values in UK10K were 9 Mb (5–16) for both parameters.

### Novel variants

We identified a total of 108 615 (37%) novel variants in the Vis exome sequence data that were not present in UK10K-GS, 136 617 (47%) novel variants not present in NHLBI, 114 176 (39%) novel variants not present in ExAC, 60 345 (21%) novel variants not present in the 1000Genomes, 34 497 (12%) novel variants not present in dbSNP and a total of 25 430 (9%) novel variants not present in any of the above mentioned datasets (Supplementary Figure 5). The count of novel variants by MAF for comparisons of Vis exome data with UK10K-GS, NHLBI, ExAC, 1000Genomes and dbSNP (Supplementary Table 4) clearly shows that the majority of novel variants belong to the rare allele frequency spectrum (up to MAF 0.02).^1^ As expected, the greatest number of singletons and doubletons are found in dbSNP (78%), followed by comparison with ExAC (58%), then 1000Genomes (54%), NHLBI (43%) and UK10K-GS (31%). A larger proportion of common allele novel variants (~40% of all common variants) was identified through comparisons with NHLBI or ExAC datasets as these variants are mostly intronic (⩾69%) and are simply not present in datasets, which are limited to exome sequence only. We can also see that there are very few common novel variants identified through comparison with 1000Genomes (0.01%) and dbSNP (0.001%), which are databases with a very good capture of common variation (Supplementary Table 4). Owing to the intronic regions within the exome calling for the Vis sample, the largest effect category group of novel variants in all five comparisons is intronic, followed by either non-synonymous (UK10K-GS, 1000Genomes, dbSNP) or non-coding RNA (NHLBI, ExAC) groups (Supplementary Figure 5). A detailed presentation of the number of both summarised and the full set of functional effects of novel variants by MAF can be found in Supplementary material (Supplementary Tables 5 and 6; Supplementary Figures 6 and 7).

**Figure 2:**
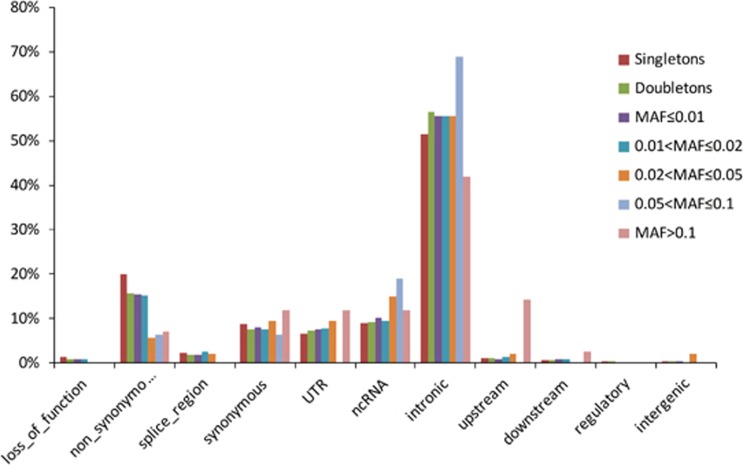
Proportion of functional effects of completely novel variants by MAF.

A detailed view of the allele frequency/functional effect distributions of completely novel variants (Table 1 and Figure 2) show that 79% of these were singletons and an additional 17% were rare or low frequency, highlighting the ability of WES to identify rare and low-frequency variants. On the contrary, only 59 completely novel variants (0.23%) were common suggesting that the discovery of a large number of common novel variation through exome sequencing is highly unlikely. Of completely novel variants, 52% were intronic, 18% were non-synonymous, whereas percentages of non-coding RNA, synonymous and UTR variants were comparable (from 7 to 9%) indicating that in comparison to distribution of all variants, number of completely novel synonymous variants is substantially decreased (Figures 1 and 2).

### Loss of function variants

The average number of LoF genotypes per genome in Vis and UK10K-GS samples was 115 (range, 93–140) and 122 (96–149), respectively; whereas the corresponding number of LoF variants in homozygous state: 23 (15–34) and 24 (13–37), was comparable between the datasets. On average, an individual in Vis had lower counts of LoF genotypes by 6% (mean difference of 6.5 genotypes per genome, 95% CI: 5–8, *P*=3.3 × 10^−16^), LoF variants by 5% (7.7, 95% CI: 6–9, *P*=6.6 × 10^−16^) and homozygous LoF variants by 5% (1.3, 95% CI: 0.6–2.0, *P*=3.1 × 10^−4^) than in UK10K-GS.

In comparison to the dataset of high confidence LoF SNVs (*n*=880) published by MacArthur *et al.*^15^, we identified 172 overlapping LoF SNVs in Vis and 227 in UK10K-GS dataset. Using only these SNVs, the estimated number of potentially harmful LoF genotypes per individual was 28 (17–39) in Vis and 29 (17–39) in UK10K-GS, and the corresponding number of homozygous high confidence LoF variants was 5 (1–12) and 6 (0–14).

As expected, the largest proportion of LoF variants in Vis belonged to the singleton category, whereas the proportions of doubleton, low frequency and common variants were comparable (Supplementary Figure 2a). Compared to LoF variants in UK10K_GS sample, there was a depletion of LoF singletons (two-proportion *z*-test, *P*=0.002), overall rare LoF variants (*P*=1 × 10^−6^) in Vis, and an excess of low frequency (*P*=1 × 10^−5^) and common variants (*P*=0.016) (Supplementary Figure 2b). A similar finding is observed when we compared allele frequency distributions of all variants shared by the two datasets or when we re-sampled the same number of individuals from each population and compared distributions of shared LoF variants.

Of the mapped genes containing LoF variants (*n*=1451), 1355 and 786 genes were found in at least one GO-term category or one pathway, respectively. Pathways that were significantly over-represented included 52 identified genes, of which the vast majority were related to xenobiotic- (metabolization or activation; 27 genes or 52%) and/or to lipid metabolism (30 genes or 58%) (Table 2). The most significant over-represented GO terms were: ion binding (GO:0043167), hydrolase (GO:0016787) or oxidoreductase activity (GO:0016491), with most genes assigned to these three categories being associated with enzyme activity (61%).

We analysed in more detail a subclass of LoF variants exhibiting high-MAF variability across Vis and the 1000Genomes Project super populations. In total, we identified nine LoF variants belonging to this subclass (Table 3). Unlike the majority of LoF variants that are expected to be rare and to have limited geographic distribution, the allele frequency of these variants ranged from rare to common across different populations.

### LoF variants in ROH regions

The distribution of LoF variants across an exome with respect to ROH hotspots and coldspots regions has shown an increase in frequency of LoF variants in hotspots, which was significant at *α*=0.1 (OR 1.18, *P*=0.083). The pathway and GO-term analyses of genes containing the hotspot LoF variants have shown intriguing results. Of the 92 mapped genes carrying a LoF variant in a ROH hotspot region, 59% were assigned to over-represented GO terms, all of which were exclusively membrane-related, either by the location, or the membrane-related function or process (Table 4). Moreover, with regard to processes or functions assigned to these terms, as a rule they were included in some sort of cell's response to either internal or external signal. Similar was found with the pathway analysis, which showed that of 48 mapped genes 53% belonged to the over-represented pathways that were mainly related to cell's response to environmental signals, such as xenobiotics, odorant molecules or allografts. Majority of identified genes in over-represented families (76%) belonged to cytochrome P450 (CYP) or solute-carrier (SLC) gene superfamilies, olfactory receptors (OR) or to HLA gene family.

## Discussion

The purpose of this work was to provide a comprehensive insight into the exomic structure of the isolated population of the Adriatic island of Vis and to compare it with the UK10K-GS exome dataset, and with reference databases (NHLBI, 1000Genomes, dbSNP and ExAC). Our data support the findings that the population of Vis is a true genetic isolate. In comparison with the UK10K-GS exomes, we see a depletion of rare and an excess of low frequency and common variants, which is suggesting that the population of Vis had undergone a bottleneck in which the majority of rare variants have vanished but those that did stay in the population have risen in frequency. We see there is a burden of potentially deleterious variants in a low and common allele frequency groups in Vis compared to UK10K-GS (Supplementary Figure 2b). This is in line with several recent studies that identified an excess of deleterious variants in the low-frequency range.^16, 17, 18, 19, 20^ Also, it was already shown that there are more deleterious variants in European than in African populations due to a long bottleneck effect during out of Africa migrations.^21^ Finally, although only unrelated individuals were included in our study, when Vis sample was compared to the UK10K-GS sample, it had higher prevalence of individuals affected with long ROH regions and had higher F_ROH >5Mb_ index. In fact, the value of F_ROH_ index in Vis was similar to the value observed for unrelated sample of Orkney inbread population.^14^

Recent population genetics studies indicate that rapid growth increases the load of rare variants^22, 23^ and likely plays a role in the individual genetic burden.^23^ Furthermore, it was demonstrated in a simulation study that population growth dramatically increases the number of deleterious sites in the population and increases the deleterious burden carried by each individual by ~6%.^24^ Following these findings, one might expect that a population with slower population growth may have a relatively smaller load of rare variants and deleterious sites. Thus, the slightly lower average number of LoF variant genotypes per Vis genome compared to UK10K-GS genome could, at least in part, be due to limited population growth of the old isolate on Vis,^3^ compared to the UK10K-GS population.

Estimates on average abundance of LoF genotypes per genome in Vis (115) or UK10K-GS (122) were somewhat larger than the value of 110 estimated from the pilot phase of the 1000Genomes Project.^25^ The pilot-phase value was based on a highly curated LoF variant list and assessed around 100 LoF variants per individual with ~20 variants in homozygous sites,^15^ suggesting slight over estimation of individual number of LoF genotypes in our sample, possibly due to a less strict filtering procedure.

We detected a ~6% difference in the average per genome number of LoF variant genotypes between Vis and UK10K-GS. This small difference persisted (~6%) after singleton and doubleton variants (those most affected by differences in sample size^22^ and also the most abundant LoF variants) were removed in order to investigate the effect of sample size differences on estimates of individual genetic burden in Vis (*n*=176) and UK10K-GS (*n*=377).

Genes containing LoF variants in Vis were over-represented in the xenobiotic- and lipid-metabolism pathways. Having in mind that the identified lipid pathways are actually involved in the biosynthesis of known endogenious xenobiotics (steroids, eicosanoids, fatty acids), over-represented genes in Vis were almost entirely xenobiotic-related suggesting that LoF mutations may be accumulating in genes controlling the cell's reaction to foreign molecules from the environment. The finding is in line with a most recent view that gene losses are not necessarily evolutionary disadvantages, but can also contribute to selective advantage in humans.^26^ In bacteria, in constant nutrient-limited environment, LoF mutations have been shown to enhance fitness by disproportionately affecting enzymatic and regulatory pathways,^27^ similar to what was found in our study. In addition, over-represented genes found within ROH hotspot regions in Vis were predominantly involved in some sort of external or internal signalling. The disruption of signalling networks responsible for regulating the response to environmental changes was identified in yeast as the major theme of adaptive evolution in constant environments.^28^ Whether the same adaptive evolutionary process could also be used to explain the evolution of Vis population given the constant physical (geography and climate) and nutrient-limited environment should be investigated further.

As the majority of LoF variants are rare and expected to have very limited geographic distribution,^29, 30^ we were particularly interested in the subclass of putative LoF variants exhibiting extremely high variability of allele frequency across populations, ranging from rare to MAF. We identified nine LoF variants with marked geographical differences which are, therefore, probable targets of positive selection.^31^ Most of the variants were associated with proteins involved in immune/defence response to pathogens (FGR-regulator of immune response, CD300LD-immune receptor, NAE1-a protooncogen involved in regulation of apoptosis, CENPM-centromere protein which also encodes a human minor histocompatibility antigen, GAS8-cytoskeletal linker, a putative tumour suppressor also implicated in influenza virus release); or proteins that are potential targets for pathogens to infect cells (that is, glycosylated extracellular membrane proteins, such as MAGEE2-a member of tumour specific antigen family and TXLNB gene suspected of role in vesicle traffic) (Table 3). In addition, four (44%) of these nine variants were also assigned in the ENSEMBLE GENE e72 database as ‘unclassified regulatory features', that is, regulatory variants that could be involved in gene transcription regulation.

A primary purpose of this study was also to catalogue variants that were not previously found in reference databases (UK10K-GS, NHLBI, 1000Genomes and dbSNP). We found 9% of all variants in Vis to be completely novel. Unsurprisingly, the vast majority of completely novel variants were singletons, rare or low frequency. The most common functional effect categories were intronic variants, followed by non-synonymous and synonymous. An interesting observation is that the number of SNVs found in dbSNP (88%) was lower than anticipated for a European sample.^32^ With most variants already catalogued in dbSNP and/or by 1000Genomes, it is expected for a single European sample that more than 90% of true variants are identified in the dbSNP database.^32^ When we took into account the rate of completely novel variants, that is, those not found in any of the four reference datasets; the aggregate rate for known SNVs increased. Although this lower rate could be due to false positive calls, the observed *T*_i_/*T*_v_ ratio was comparable to other WES studies (Supplementary material). Alternatively, the somewhat lower dbSNP rate might be a consequence of two main factors: background population and demographic history. Our study samples were taken from an isolated island population where we expect a drift up in the frequency of rare variants unique to our sample, which could result in increased proportion of detected novel variants.

Complete identification of all human variants is one of the key goals of modern genetics. This study represents a step forward in this major challenge by providing a population-based catalogue of variants and, importantly, identifying completely novel exomic variants in the population isolate from the Adriatic island of Vis. It also reveals the landscape of loss-of-function mutations that is intriguing in terms of adaptive evolution. By providing a relatively large set of variants not seen elsewhere, this study serves as a valuable resource in understanding human variation, especially in the light of genetic studies of human diseases, population genetics and evolution in this population.

## Figures and Tables

**Table 1:** The count of all variants, and completely novel variants in each functional effect group separated by allele frequency categories

*Variant consequence annotations*	*All variants*	*Singletons*	*Doubletons*	*MAF*⩽*0.01*	*0.01<MAF*⩽*0.02*	*0.02<MAF*⩽*0.05*	*0.05<MAF*⩽*0.1*	*MAF>0.1*
*All variants*								
Loss_of_function	1775	892	219	100	152	93	79	240
Non_synonymous	60 011	22 939	6988	3626	6119	4834	3944	11 561
Splice_region	8748	2657	886	468	892	791	689	2365
Synonymous	44 582	12 891	4292	2501	4491	4111	3658	12 638
UTR	17 165	4945	1792	1073	1865	1585	1438	4467
ncRNA	23 033	6292	2210	1256	2274	2190	2145	6666
Intronic	131 531	35 463	13 016	7519	13 797	12 426	11 828	37 482
Upstream	2056	506	206	118	204	181	209	632
Downstream	1142	265	108	63	105	114	131	356
Regulatory	73	13	8	5	8	5	11	23
Intergenic	461	106	28	18	43	38	50	178
Total	290 577	86 969	29 753	16 747	29 950	26 368	24 182	76 608
								
*Completely novel variants*								
Loss_of_function	269	230	27	7	5	0	0	0
Non_synonymous	4783	3978	515	167	116	3	1	3
Splice_region	552	455	60	17	19	1	0	0
Synonymous	2141	1738	250	85	57	5	1	5
UTR	1702	1312	240	81	59	5	0	5
ncRNA	2273	1772	305	109	71	8	3	5
Intronic	13 308	10 328	1890	604	427	30	11	18
Upstream	230	175	32	7	9	1	0	6
Downstream	120	86	19	8	6	0	0	1
Regulatory	9	6	3	0	0	0	0	0
Intergenic	44	38	3	2	0	1	0	0
Total	25 431	20 118	3344	1087	769	54	16	43

**Table 2:** Genes with LoF variants — summary of predicted gene product function and location using gene ontology terms and pathway analysis

*Pathway name*	*Pathway source*	*Total set size*	*No. of identified genes*	P*-value*	*q-value*	*Description*
*Over-represented pathways*^a^						
CYP2E1 reactions	Reactome	11	6 (54.5%)	0.00004	0.0518	Xenobiotics related
Leukotriene metabolism	EHMN	104	19 (18.3%)	9.89 × 10^−5^	0.0518	Lipid metabolism
Galactose metabolism	KEGG	30	9 (30.0%)	0.000148	0.0518	Other
Tryptophan degradation	INOH	66	14 (21.2%)	0.000158	0.0518	Xenobiotics related^b^
Metabolism of xenobiotics by cytochrome P450	KEGG	74	15 (20.3%)	0.000159	0.0518	Xenobiotics related
Androgen and oestrogen biosynthesis and metabolism	EHMN	87	16 (18.4%)	0.000318	0.0789	Lipid metabolism
Fatty acids	Reactome	15	6 (40.0%)	0.000339	0.0789	Lipid metabolism
Xenobiotics	Reactome	21	7 (33.3%)	0.000398	0.0803	Xenobiotics related
Chemical carcinogenesis	KEGG	81	15 (18.5%)	0.000448	0.0803	Xenobiotics related
C21-steroid hormone biosynthesis and metabolism	EHMN	57	12 (21.1%)	0.000493	0.0803	Lipid metabolism

*GO term*	*Total set size*	*No. of identified genes*	P*-value*	*q-value*
*Over-represented GO terms*^b^				
*GO group: Molecular function*				
GO:0043167 ion binding	6117	531 (8.7%)	8.85 × 10^−8^	4.6 × 10^−6^
GO:0016787 hydrolase activity	2477	235 (9.5%)	3.66 × 10^−6^	9.51 × 10^−5^
GO:0016491 oxidoreductase activity	728	77 (10.6%)	0.000529	0.00917
GO:0019825 oxygen binding	47	9 (19.1%)	0.00601	0.071
GO:0030246 carbohydrate binding	279	32 (11.5%)	0.00683	0.071
GO:0004601 peroxidase activity	41	8 (19.5%)	0.00836	0.0724
*GO group: Cellular compartment*				
GO:0005929 cilium	465	53 (11.4%)	0.000719	0.0302
GO:0044441 ciliary part	309	38 (12.3%)	0.000987	0.0302
GO:0005578 proteinaceous extracellular matrix	356	42 (11.9%)	0.00113	0.0302
GO:0034358 plasma lipoprotein particle	39	8 (20.5%)	0.00612	0.0926
GO:0005615 extracellular space	1338	120 (9.0%)	0.00686	0.0926
GO:0072562 blood microparticle	135	18 (13.4%)	0.00821	0.0926
GO:0032994 protein–lipid complex	41	8 (19.5%)	0.00836	0.0926
GO:0005604 basement membrane	97	14 (14.6%)	0.00926	0.0926

a Annotations are ordered by *q*-values.

b Tryptophan degradation pathway is classified as xenobiotic-related as tryptophan metabolites are known to activate aryl hydrocarbon receptor, transcription factor known to mediate most of the toxic and carcinogenic effects of a wide variety of environmental contaminants.

**Table 3:** Putative LoF variants (*n*=9) with extremely high variability among populations: Vis and 1000Genomes super populations EUR, ASN, AFR, AMR

					*Allele frequency in population*					
*CHR*	*Position (bp)*^a^	*ID*	*REF*	*ALT*	*Vis*	*EUR*	*AMR*	*AFR*	*ASN*	*Functional annotation*^b^ *Gene/regulatory element ID, functional annotation, number of transcripts;*
1	27942176	rs2231879	T	C	0.02	0.02	0.07	0.51	—	Regulatory element ID: ENSR00001518649, regulatory_region_variant, — Gene ID: FGR, splice_acceptor_variant, nc_transcript_variant, 1; intron_variant, 6
5	111481696	rs17134155	C	T	0.18	0.18	0.13	0.52	0.05	Regulatory element ID: ENSR00001287518, regulatory_region_variant, — Gene ID: EPB41L4A, splice_acceptor_variant, nc_transcript_variant, 1
6	139576544	rs41289819	G	A	0.13	0.16	0.14	0.53	0.02	Gene ID: TXLNB, stop_gained:373:125, 1; intron_variant, 1
7	144364918	rs67644764	G	T	0.06	0.05	0.11	0.61	0.002	Gene ID: TPK1, stop_gained:71:24, 1; intron_variant, NMD_transcript_variant, 2; intron_variant, 3; intron_variant, nc_transcript_variant, 1; upstream_gene_variant, 2; synonymous_variant, NMD_transcript_variant:129:43:L>L, 1; 5_prime_UTR_variant, 1
16	66861836	rs7195853	G	A	0.05	0.07	0.09	0.56	0.03	Gene ID: NAE1 splice_donor_variant, NMD_transcript_variant, 1; intron_variant, NMD_transcript_variant, 3; intron_variant, 8; intron_variant, nc_transcript_variant, 4;
16	90110950	rs1048149	C	T	0.12	0.14	0.19	0.59	0.03	Regulatory element ID: ENSR00000512444, regulatory_region_variant, — Gene ID: ENSG00000222019, stop_gained:68:23, 2; stop_gained,NMD_transcript_variant:68:23, 1; non_coding_exon_variant, nc_transcript_variant, 1 Gene ID: GAS8, 3_prime_UTR_variant, NMD_transcript_variant, 1; 3_prime_UTR_variant, 1; downstream_gene_variant, 5; non_coding_exon_variant,nc_transcript_variant, 1
17	72588806	rs545652	C	A	0.11	0.14	0.19	0.52	0.04	Gene ID: C17orf77, stop_gained:621:207, 2; downstream_gene_variant, 1 Gene ID: CD300LD, upstream_gene_variant, 1
22	42336172	rs5758511	G	A	0.25	0.27	0.20	0.03	0.51	Regulatory element ID: ENSR00000085774, regulatory_region_variant, — Gene ID: CENPM stop_gained:7:3, 1; intron_variant, 5; downstream_gene_variant, 1
X	75004529	rs1343879	C	A	0.02	0.03	0.24	0.05	0.91	Gene ID: MAGEE2, stop_gained:358:120, 1

a The genomic reference sequence used is GRCh37/hg19. Population allele frequency of variants range from rare to common major allele.

b Called with the Ensembl Variant Effect Predictor v2.8 against Ensembl 70.

**Table 4:** Genes with LoF variants in ROH hotspots—summary of predicted-gene product function and location using gene ontology terms and pathway analysis

*Pathway name*	*Pathway source*	*Total set size*	*No. of Identified genes*	P*-value*	*q-value*	*Description*
*Over-represented pathways*^a^						
Allograft rejection	Wikipathways	80	5 (6.2%)	2.21 × 10^−5^	0.00225	Immune response to allograft
Cytochrome P450—arranged by substrate type	Reactome	61	4 (6.6%)	0.00013	0.00662	Xenobiotics metabolism
Phase 1—functionalization of compounds	Reactome	79	4 (5.1%)	0.000353	0.00882	Xenobiotics metabolism
Olfactory signalling pathway	Reactome	427	8 (1.9%)	0.000401	0.00882	Response to external signal
Warfarin pathway, pharmacokinetics	PharmGKB	8	2 (25.0%)	0.000496	0.00882	Xenobiotics metabolism
Allograft rejection—*Homo sapiens* (human)	KEGG	37	3 (8.1%)	0.000519	0.00882	Immune response to allograft

*GO term*	*Total set size*	*No. of identified genes*	P*-value*	*q-value*	*Description*
*Over-represented GO terms*^a^					
*GO group: Cellular component*					
GO:0098805 whole membrane	1916	20 (1.0%)	0.000295	0.00534	Membrane location
GO:0098576 lumenal side of membrane	30	3 (10.3%)	0.000305	0.00534	Membrane location
GO:0098589 membrane region	1086	13 (1.2%)	0.00119	0.0138	Membrane location
GO:0005886 plasma membrane	4864	34 (0.7%)	0.00315	0.0276	Membrane location
GO:0071944 cell periphery	4967	34 (0.7%)	0.00453	0.0317	Membrane location
GO:0048475 coated membrane	88	3 (3.4%)	0.00756	0.0362	Membrane location
GO:0031300 intrinsic component of organelle membrane	271	5 (1.9%)	0.00781	0.0362	Membrane location
*GO group: Molecular function*					
GO:0044459 plasma membrane part	2528	20 (0.8%)	0.00829	0.0362	Membrane location
GO:0005549 odorant binding	85	4 (4.7%)	0.000612	0.0134	Binding—external signal
GO:0003823 antigen binding	103	4 (4.0%)	0.00117	0.0134	Binding—external signal
GO:0004871 signal transducer activity	1663	16 (1.0%)	0.00296	0.0227	Response to internal/external signal
GO:0004872 receptor activity	1583	15 (1.0%)	0.00469	0.0269	Response to internal/external signal
*GO group: Biological process*					
GO:0045321 leucocyte activation	701	10 (1.4%)	0.00123	0.0704	Response to internal/external signal
GO:0052192 movement in environment of other organism involved in symbiotic interaction	84	3 (3.6%)	0.00665	0.099	Response to external signal
GO:0042221 response to chemical	4132	29 (0.7%)	0.0073	0.099	Response to external signal
GO:0002252 immune effector process	765	9 (1.2%)	0.00762	0.099	Response to internal/external signal

a Annotations are ordered by *q*-values.
