# Supplementary material for: Whole-exome sequencing in an isolated population from the Dalmatian island of Vis
Source: Eur J Hum Genet. 2016 Apr 6;24(10):1479–87. doi: 10.1038/ejhg.2016.23 (PMC4950961; doi:10.1038/ejhg.2016.23)
Supplement: Supplementary Information [file ejhg201623x1.doc]

**Supplementary material**

Contents

[Variant calling 1](#__RefHeading___Toc420993770)

[Ti/Tv ratio 1](#__RefHeading___Toc420993771)

[Quality control - per sample and variant site 2](#__RefHeading___Toc420993772)

[Tables 4](#__RefHeading___Toc420993773)

[Figures 11](#__RefHeading___Toc420993774)

[References 22](#__RefHeading___Toc420993775)

## Variant calling

Sequence reads were aligned with the Burrows-Wheeler Aligner (BWA) algorithm to the NCBI human reference genome (build 37). Samtools 0.1.19 mpileup was used to sort, merge, and manipulate aligned sequence files and create a ‘pileup’ of reads for each sample. Duplicates were marked with Picard and local re-alignment and base quality score recalibration were carried out using Genome Analysis Toolkit (GATK). After data pre-processing, variant callings combined across samples were performed.

Multi-sample variant-calling was performed using Samtools mpileup v 0.1.19, followed by VQSR filtering of single nucleotide variants (SNVs) and indels. SNVs were filtered by minimum VQSLOD score corresponding to the truth sensitivity threshold of 99.5% and genotype filtering was utilized if the particular call fell outside given ranges: depth between 4 and 2000, and genotype quality between 20 and 60.

## Ti/Tv ratio

To assess the probability of false positives we also evaluated aggregate transition transversion (Ti/Tv) ratio for on-target variants. Average Ti/Tv ratio for targeted regions over all subjects and all locations was 2.9 with the ratio somewhat increased for known (3.1), and decreased for novel (2.4) on-target variants. Most of the transversions in the novel on-target variant set originated from rare mutations, notably singletons and doubletons (87%). After elimination of these variants, Ti/Tv ratio for novel on-target variant increased to 2.6. Comparable results were obtained on UK10K-GS data: overall on-target Ti/Tv ratio of 3.03, 3.21 for known, and 2.73 for novel on-target variants with singletons and doubletons being responsible for 94% of all transversions in novel on-target set. As implied by the aggregate on-target Ti/Tv ratio, the calling pipeline produced a good quality variant call set.

The value of 2.9 for aggregate on-target Ti/Tv ratio was close to the range of Ti/Tv ratio from 3.0 to 3.5 that was estimated from whole-exome call sets.[^1^](#_ENREF_1) Given that target regions also included non-coding variants (ie. in small non-coding RNA regions) the observed ratio was closer to the lower boundary of the range. A similar ratio of 3.0 was also observed in UK10K-GS variant call set, additionally supporting the feature of target regions, and not sequencing errors, as predominant factor affecting Ti/Tv metric. The difference in Ti/Tv ratio between on-target known vs. novel variants (3.1 *vs* 2.4) was likely due to increased proportion of intronic variants in on-target novel variants.

## Quality control - per sample and variant site

The sample-level QC criteria were: a) genotype concordance for the overlapping set of individuals/SNVs between Vis genome-wide data (genotyped using Illumina HumanHap300-Duo BeadChip) and exome-sequence was examined resulting with a very high genotype concordance rate (mean 99.6%, stdev 0.6%) (Supplementary Figure 1); b) all samples passed the sample call rate exclusion threshold of 90%; c) heterozygosity exclusion threshold of ±3standard deviations (SD) from the mean was applied; d) heterozygosity to homozygosity ratio was evaluated e) multidimensional scaling analysis (MDS) was performed, plots were visually inspected and no subjects of different ancestry than CEU were found; f) identity by state (IBS)/ identity by descent (IBD) statistics were calculated for identification of duplicated and related samples. An exclusion threshold for IBD, pi-hat greater than 0.2, was used; g) singleton count per sample was calculated and exclusion threshold of ±3 SD from the mean was applied. All samples failing sample-level QC were removed within each dataset prior to performing SNV-level QC.

The SNV-level QC step was carried out under the following criteria: a) SNV call rate threshold of 99% for rare and low frequency alleles (minor allele frequency (MAF) < 0.05) and 95% for common alleles (MAF ≥0.05) were applied; b) deviation from Hardy-Weinberg equilibrium (HWE) was calculated and an exclusion list of SNVs falling below threshold for HWE, exact p<1x10^-4^, was generated. All SNVs that failed individual SNV-level QC steps were removed. All subsequent analyses were performed using clean post-QCed datasets.

QC steps were performed using PLINK.[^2^](#_ENREF_2) The post-QCed Vis exome-sequence dataset contained 176 individuals and 290,577 SNVs, while the UK10K-GS dataset contained 377 individuals and 398,743 SNVs.

## Tables

**Supplementary Table 1:** The full list of Ensembl consequence annotations and their grouping into fewer categories.

**Supplementary Table 2:** Putative LoF variants (n=24) excluded from further analysis as likely reference error due to unchanged major allele status across all examined populations: Vis and 1KG super populations

|  |  |  |  |  | Allele frequency | | | | |
| --- | --- | --- | --- | --- | --- | --- | --- | --- | --- |
| CHR | Position [bp] | ID | ALT | REF | VIS | EUR | AMR | AFR | ASN |
| 1 | 5935162 | rs1287637 | T | A | 0.81 | 0.81 | 0.81 | 0.88 | 0.83 |
| 1 | 46496709 | rs925524 | G | A | 0.70 | 0.74 | 0.67 | 0.59 | 0.69 |
| 2 | 240323661 | rs1709851 | A | C | 0.63 | 0.65 | 0.75 | 0.73 | 0.94 |
| 3 | 113012797 | rs810209 | A | G | 0.73 | 0.76 | 0.81 | 0.51 | 0.74 |
| 6 | 31239722 | rs9264670 | C | A | 0.91 | 0.85 | 0.88 | 0.89 | 0.80 |
| 6 | 32411035 | rs8084 | C | A | 0.65 | 0.56 | 0.64 | 0.58 | 0.67 |
| 7 | 64438667 | rs1404453 | A | G | 0.94 | 0.96 | 0.94 | 0.73 | 0.88 |
| 8 | 99205612 | rs3735887 | T | C | 0.52 | 0.51 | 0.63 | 0.55 | 0.60 |
| 8 | 100133706 | rs7460625 | G | T | 0.80 | 0.77 | 0.64 | 0.67 | 0.53 |
| 8 | 142505038 | rs6578193 | C | T | 0.79 | 0.77 | 0.83 | 0.84 | 0.65 |
| 9 | 136083580 | rs2073870 | G | T | 0.78 | 0.79 | 0.81 | 0.59 | 0.61 |
| 9 | 140139757 | rs9775264 | A | G | 1.00 | 0.99 | 1.00 | 1.00 | 1.00 |
| 11 | 48286231 | rs10838851 | A | T | 0.75 | 0.75 | 0.74 | 0.53 | 0.60 |
| 11 | 49000423 | rs1965370 | G | C | 0.83 | 0.85 | 0.81 | 0.82 | 0.69 |
| 11 | 62910849 | rs1939749 | C | T | 0.84 | 0.83 | 0.83 | 0.52 | 0.92 |
| 12 | 8386871 | . | G | C | 0.81 | 0.79 | 0.73 | 0.60 | 0.89 |
| 15 | 40856989 | rs3803354 | T | C | 0.89 | 0.93 | 0.91 | 0.75 | 0.90 |
| 17 | 19578873 | rs7216 | T | A | 0.55 | 0.55 | 0.62 | 0.54 | 0.98 |
| 17 | 41961451 | rs231518 | C | T | 0.86 | 0.85 | 0.90 | 0.93 | 1.00 |
| 17 | 53076986 | rs11658717 | A | G | 0.70 | 0.74 | 0.82 | 0.71 | 0.90 |
| 19 | 51920613 | rs1010425 | T | C | 0.91 | 0.88 | 0.88 | 0.78 | 0.88 |
| 19 | 57642782 | rs9973206 | A | C | 0.78 | 0.85 | 0.93 | 0.99 | 1.00 |
| 19 | 58003580 | rs2074071 | G | A | 0.70 | 0.68 | 0.71 | 0.59 | 0.72 |
| 20 | 61666063 | . | T | C | 0.62 | 0.66 | 0.66 | 0.65 | 0.77 |

The genomic reference sequence used is GRCh37/hg19

**Supplementary Table 3:** The number of variants according the full set of functional consequence annotations for each allele frequency category.

**Supplementary Table 4:** The count of novel variants by allele frequency categories for comparisons of Vis exome data with UK10K, NHLBI, 1000Genomes, dbSNP, ExAC, and completely novel variants not found in any of the five reference datasets.

**Supplementary Table 5:** The distribution of functional consequence annotations of novel variants by MAF identified through comparisons with UK10K, NHLBI GO Exome Sequencing Project, 1KG, dbSNP, ExAC, and all five altogether.

**Supplementary Table 6:** The distribution of the full set of functional consequence annotations of novel variants by MAF, identified through comparison with UK10K, NHLBI GO Exome Sequencing Project, 1KG, dbSNP, ExAC, and all five altogether.

## Figures


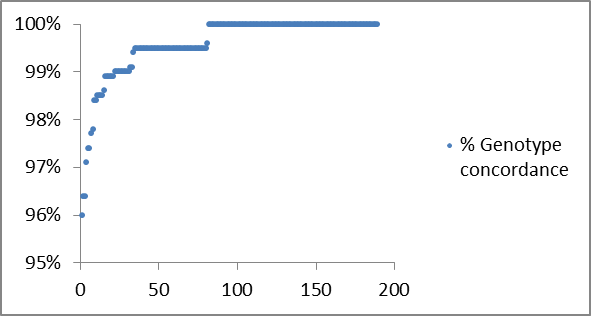


**Supplementary Figure 1:** Genotype concordance for the overlapping set of individuals/SNVs between Vis genome-wide data (genotyped using Illumina HumanHap300-Duo BeadChip) and exome-sequence data.

a)
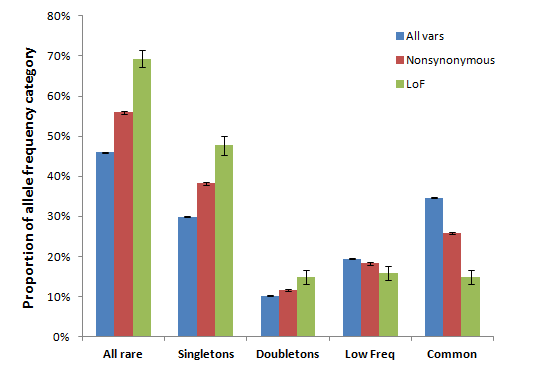


b)
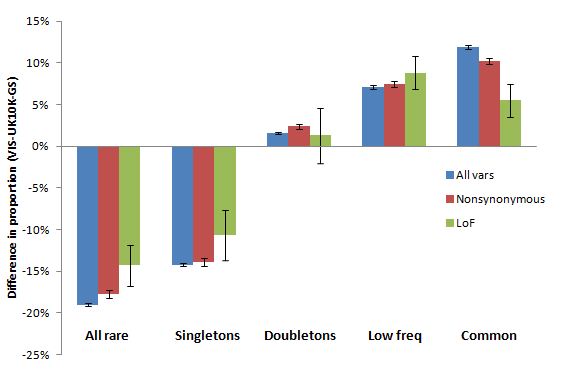


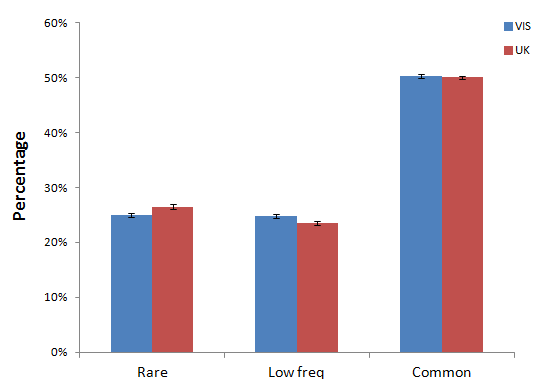


c)

**Supplementary Figure 2:** Shown, with 95% confidence interval, are: a) allele frequency distributions of all, non-synonymous, and loss-of-function variants in Vis (sample size, n=176); b) differences in proportion of variants between Vis and UK10K-GS (n=377) samples in a given allele frequency category. Also shown are: c) allele frequency distributions of variants shared by Vis and UK10K-GS datasets separated by allele frequency categories. Distributions were obtained after re-sampling the same number of individuals (n=100) from each population 100 times. Allele frequency categories were defined as: **all rare** - MAF≤0.01, **low frequency** – 0.01<MAF≤0.05, **common** – MAF>0.05


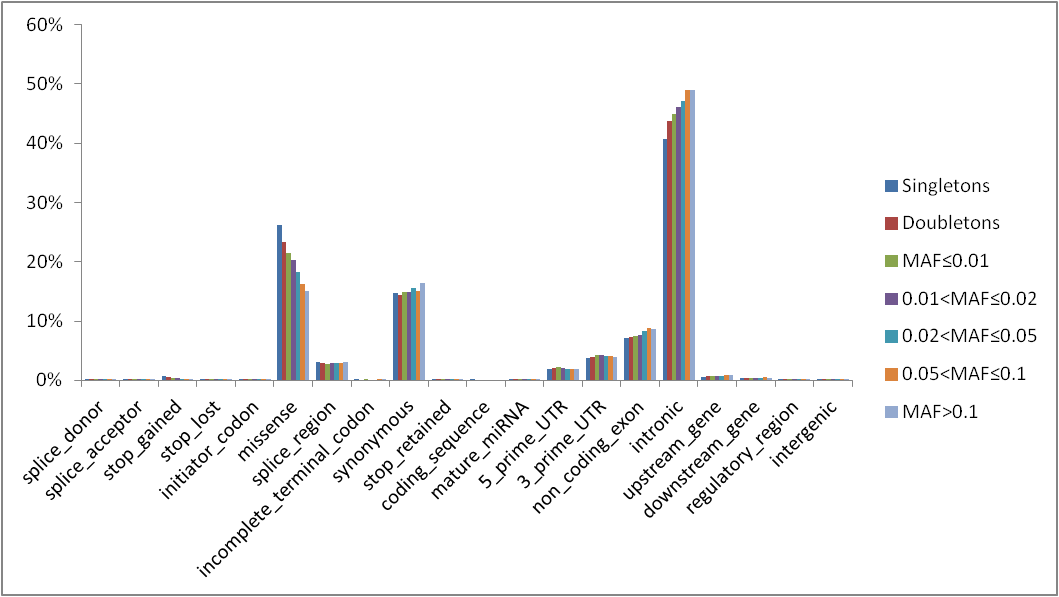


**Supplementary Figure 3:** Proportion of the full set of functional consequence annotations by allele frequency category in Vis.


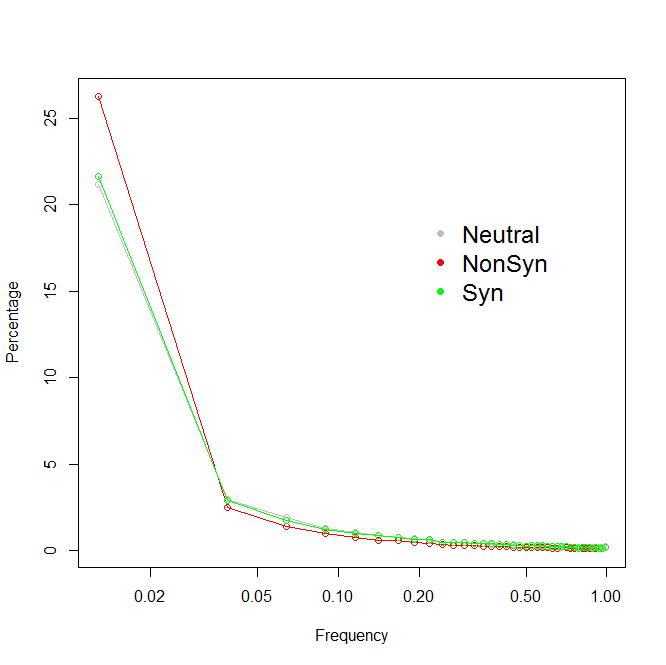


**Supplementary Figure 4:** Aggregate site frequency spectra by variant’s functional consequence, presented on a log scale.


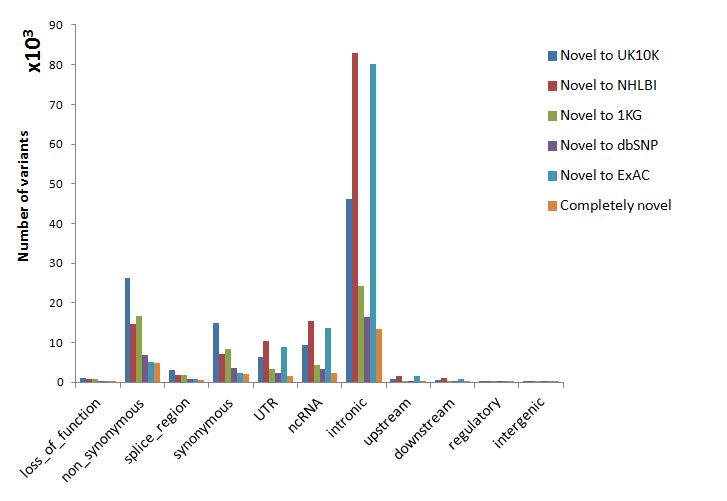


**Supplementary Figure 5:** Number of novel variants by different effect categories, comparison between Vis exome data and five reference datasets.

| 1. Variants not found in UK10K Generation Scotland |
| --- |
| 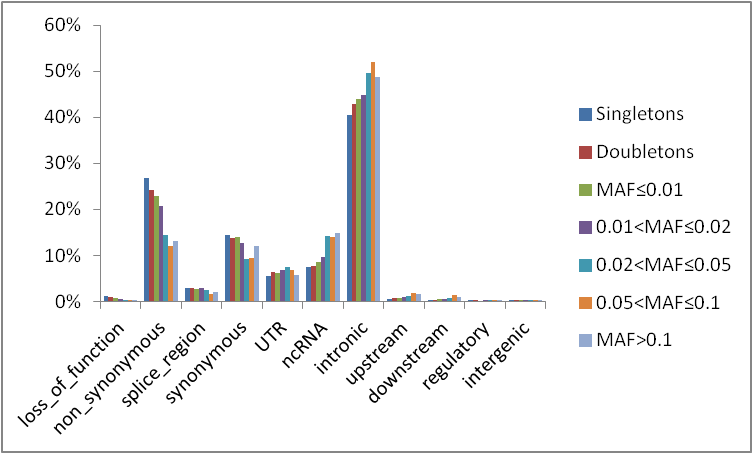 |
| 1. Variants not found in NHLBI GO Exome Sequencing Project |
| 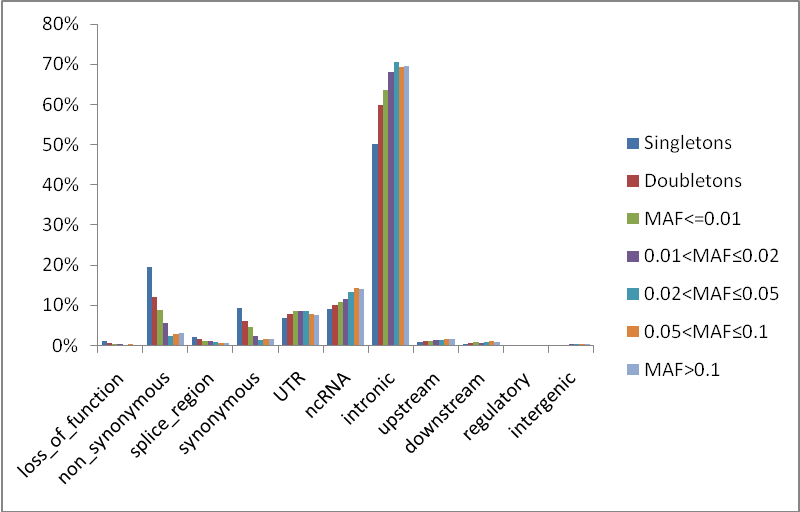 |

| 1. Variants not found in 1000Genomes project |
| --- |
| 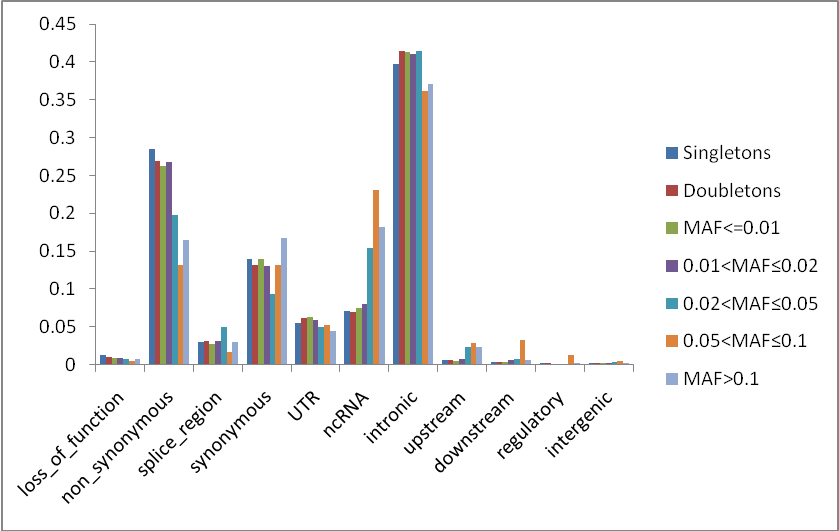 |
| 1. Variants not found in dbSNP |
| 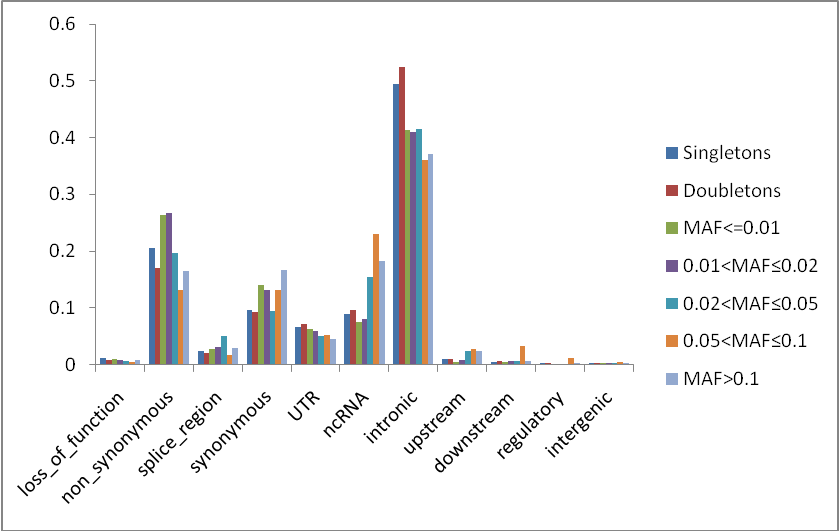 |
| 1. Variants not found in ExAC |
| 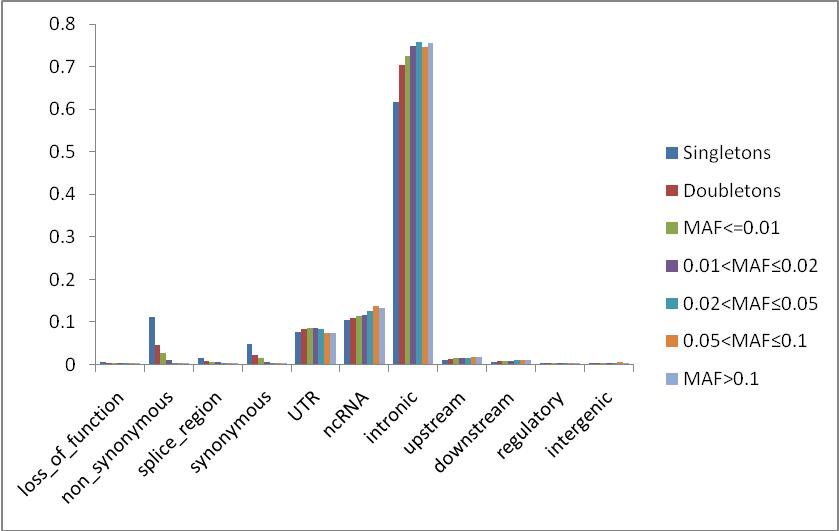 |

**Supplementary Figure 6:** Proportion of a summarised set of novel variant functional effects by MAF, identified through comparison with UK10K Generation Scotland, NHLBI GO Exome Sequencing Project, 1000Genomes, dbSNP, and ExAC.

| 1. Variants not found in UK10K Generation Scotland |
| --- |
| 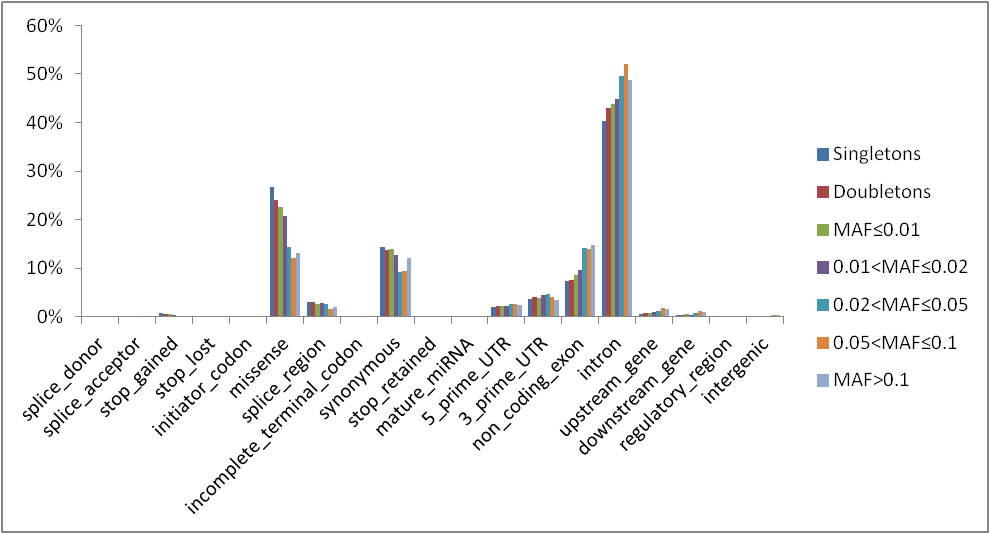 |
| 1. Variants not found in NHLBI GO Exome Sequencing Project |
| 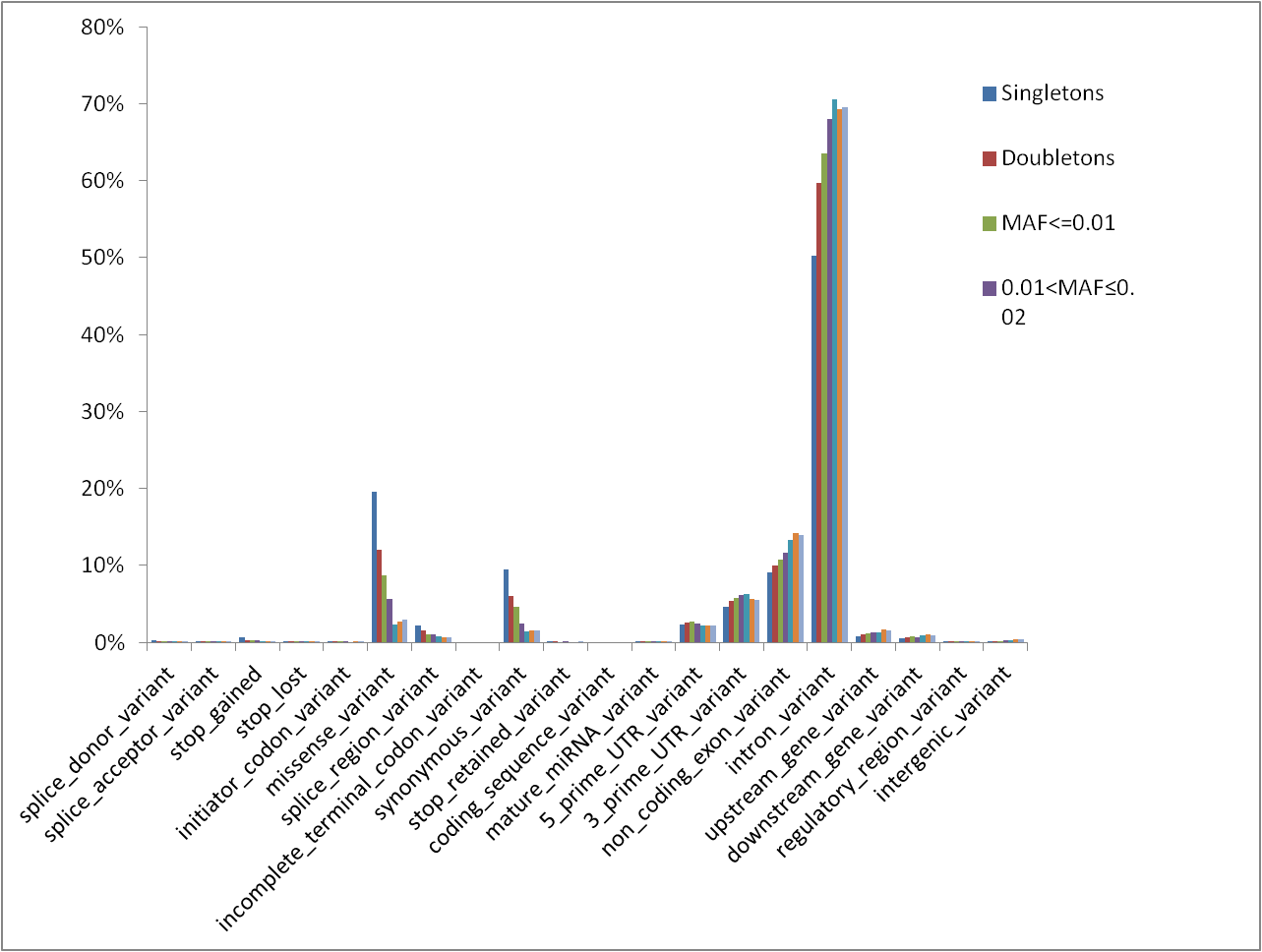 |

| 1. Variants not found in 1000Genomes project |
| --- |
| 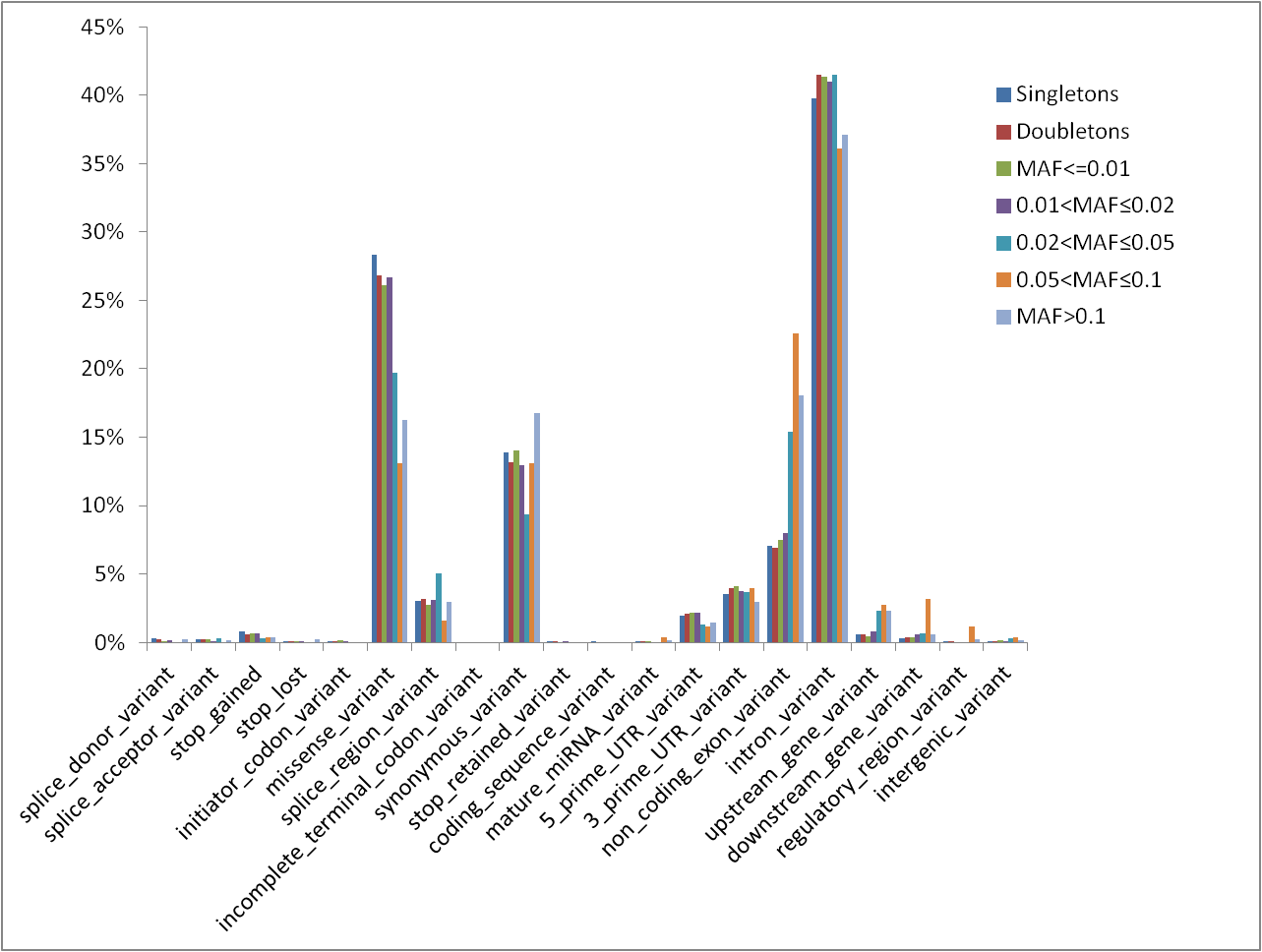 |
| 1. Variants not found in dbSNP |
| 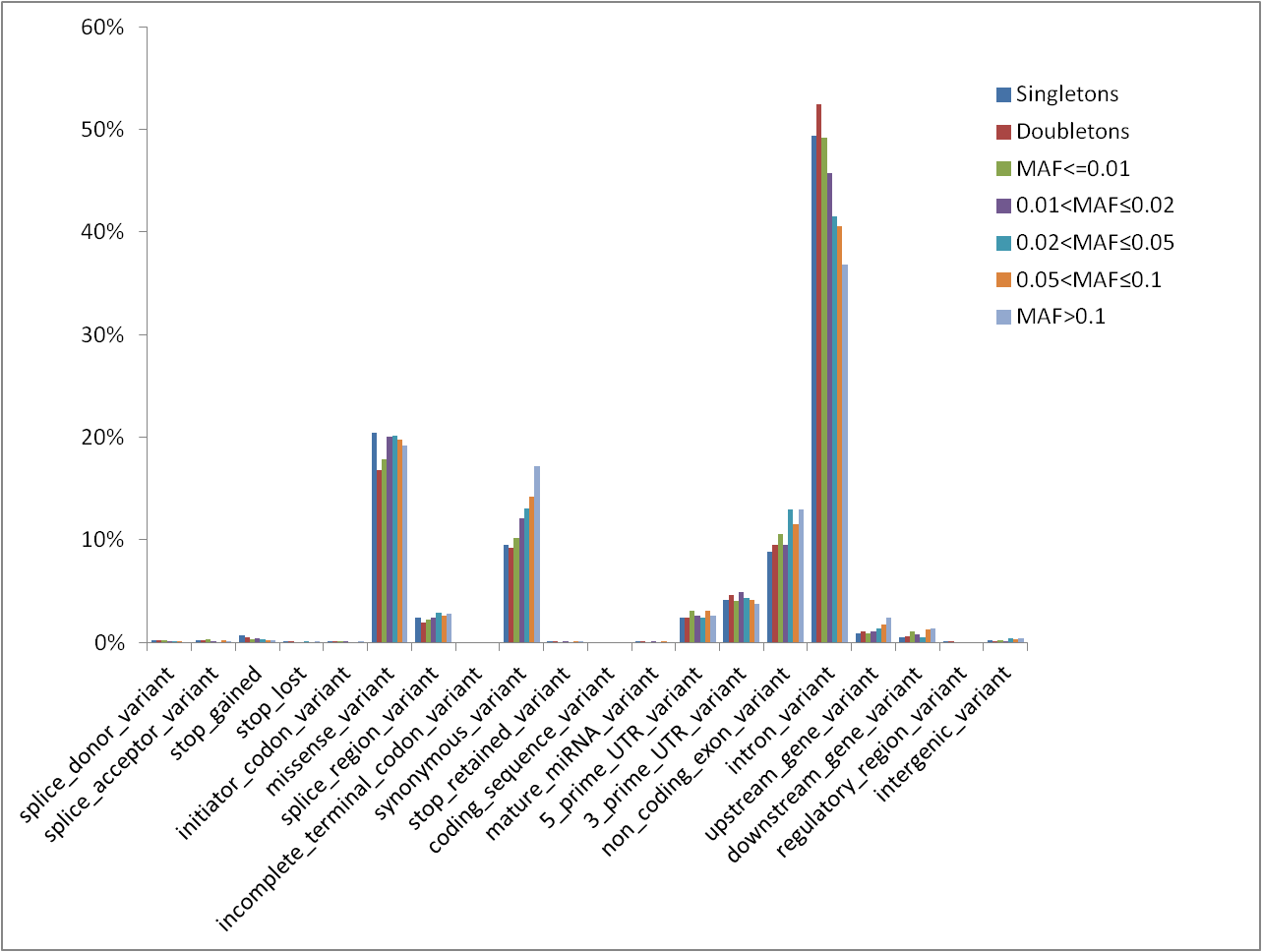 |
| 1. Variants not found in ExAC |
| 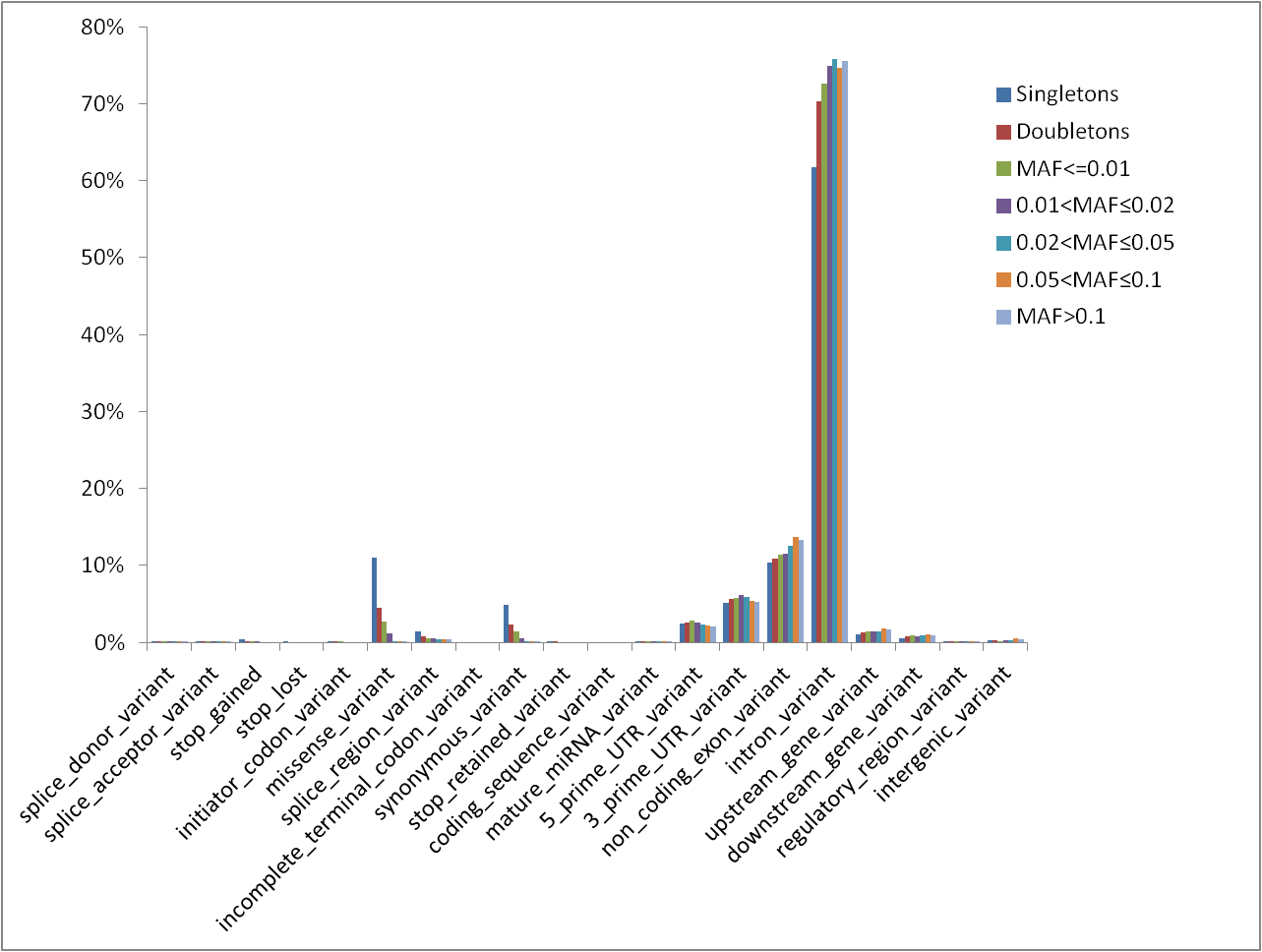 |
| 1. Variants not found in any dataset (completely novel) |
| 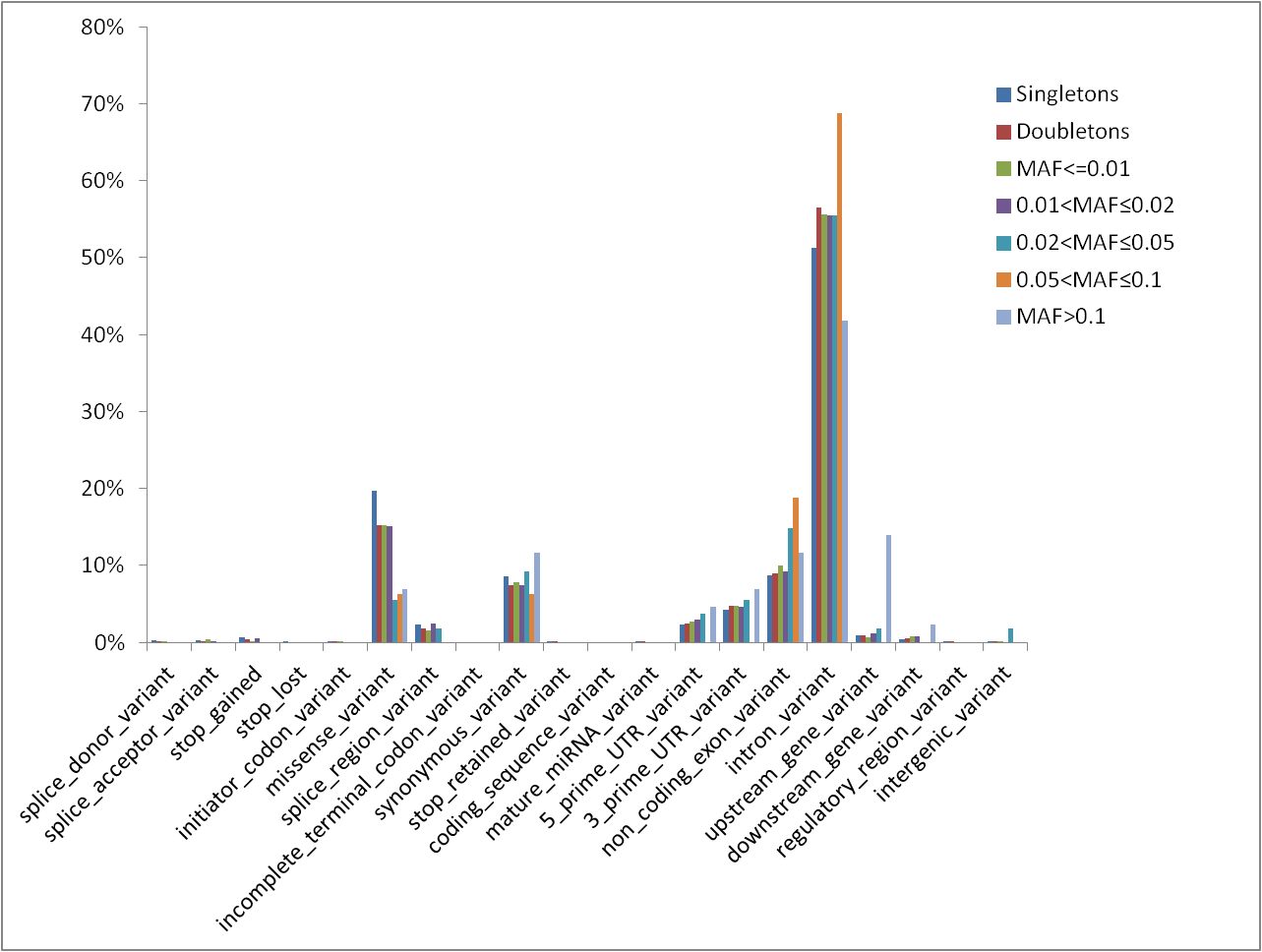 |

**Supplementary Figure 7:** Proportion of a full set of novel variant functional effects by MAF, identified through comparison with UK10K, NHLBI GO Exome Sequencing Project, 1000Genomes, dbSNP, ExAC, and all five altogether.

## References

1. DePristo MA, Banks E, Poplin R *et al*: A framework for variation discovery and genotyping using next-generation DNA sequencing data. *Nature genetics* 2011; **43:** 491-498.

2. Purcell S, Neale B, Todd-Brown K *et al*: PLINK: a tool set for whole-genome association and population-based linkage analyses. *American journal of human genetics* 2007; **81:** 559-575.
